# Clinical Evaluation of an AI-Based Prototype for Contactless Respiratory Monitoring in Children

**DOI:** 10.3390/children13020232

**Published:** 2026-02-06

**Authors:** Ludwig Maximilian Seebauer, Marcel Geis, Niklas Alexander Köhler, Claudius Nöh, Jochen Frey, Volker Groß, Keywan Sohrabi, Sebastian Kerzel

**Affiliations:** 1 Department of Pediatric Pneumology and Allergy, University Children’s Hospital Regensburg (KUNO) at the Hospital St. Hedwig of the Order of St. John, University of Regensburg, D-93049 Regensburg, Germany; sebastian.kerzel@barmherzige-regensburg.de; 2 Department of Health, University of Applied Sciences Mittelhessen, D-35390 Giessen, Germany; marcel.geis@ges.thm.de (M.G.);

**Keywords:** contactless monitoring, pediatric respiratory diseases, artificial intelligence, time-of-flight measurement, polysomnography, sleep medicine

## Abstract

Background: Pediatric respiratory disorders frequently necessitate clinical evaluation, often during sleep. Traditional polysomnography (PSG), while the gold standard for sleep-related respiratory assessment, is resource-intensive and can cause discomfort, particularly in children. Therefore, in a prior published study, we designed and technically validated a video-based prototype for contactless monitoring of respiratory movements. Objective: Our present study aimed to clinically validate the contactless monitoring prototype in pediatric patients, with a primary focus on detecting respiratory rate and identifying abnormal breathing patterns. Methods: Twenty-seven pediatric patients (aged 6 months to 12 years) were recruited from a pediatric sleep laboratory. To monitor thoracoabdominal movements in real time, the prototype employed a time-of-flight camera and a 3D imaging module, coupled with artificial-intelligence-based determination of the region of interest (ROI). Respiratory rates obtained from the prototype were compared to simultaneously recorded PSG data. Data were collected under various conditions, including different sleeping positions. A total of 296 h of respiratory data were acquired, of which selected 60 s segments (54 during N3 sleep and 27 during REM sleep) were analyzed using the prototype and compared with PSG-derived respiratory parameters. Conclusion: The contactless prototype demonstrates that reliable and non-invasive respiratory monitoring is feasible in pediatric patients. It enables accurate detection of respiratory rate as well as abnormal breathing patterns under routine clinical conditions, while reducing patient burden compared with conventional approaches. Its usability and minimal patient discomfort suggest potential for broader clinical adoption. Future work should focus on full-night recordings across all sleep stages and the development of automated data analysis pipelines to facilitate routine clinical implementation.

## 1. Introduction

Pediatric respiratory disorders are among the most common reasons for medical consultations, particularly during early childhood. Although in many cases a simple physical examination is sufficient to make the correct diagnosis, some disorders require a more detailed diagnostic workup, especially if they occur predominantly during sleep. In general, a nocturnal predominance of clinical symptoms is a hallmark of many respiratory diseases. The gold standard for assessing respiratory function during sleep is a cardiorespiratory polysomnography (PSG), which requires multiple sensors attached to the body. This often creates significant discomfort, especially for younger children, and limits its routine use to specialized clinical settings.

There has been a growing interest in developing contactless respiratory monitoring technologies that aim to reduce patient discomfort while maintaining diagnostic accuracy. Several review articles and comparative studies have summarized the current state of contactless respiratory monitoring, highlighting both its clinical potential and the remaining technical challenges, particularly in vulnerable populations such as infants and children [1,2]. Therefore, various non-contact modalities for respiratory monitoring have been explored in recent years: Infrared thermography has been utilized to detect thermal fluctuations around the nose and mouth [3]. Radar-based systems, particularly those employing continuous wave (CW) and ultra-wideband (UWB) technologies, have demonstrated the capability to detect chest movements and estimate respiratory rates with high precision [4,5]. Additionally, depth-sensing cameras have been employed to capture thoracoabdominal movements, providing valuable data on respiratory mechanics [6,7,8]. A common approach in the latter method is the use of so-called “structured light-based systems”, which detect a (more or less) distorted pattern of light cast by a projector in order to calculate the depth and shape of a target object.

Despite the promise of these technologies, each modality brings specific challenges, which have been systematically discussed in the literature with regard to robustness, motion artifacts, and suitability for clinical environments [2,9]. Infrared thermography can be affected by ambient temperature variations [3], radar systems are susceptible to motion artifacts, especially in environments with significant background movement [10], and “structured light-based systems” can be sensitive to ambient lighting conditions and surface reflectivity, potentially limiting their effectiveness in uncontrolled clinical environments [11].

To enhance the reliability of contactless respiratory monitoring, recent studies have explored the integration of multiple sensing modalities with machine learning algorithms. For instance, multimodal audio–visual approaches and machine-learning based pipelines have been proposed to improve detection of respiratory events in noisy clinical settings [12]. 

These advancements underscore the field’s progression towards integrating advanced sensor technologies with machine learning algorithms to improve feasibility while maintaining accuracy. Despite differences in sensor modality—ranging from thermal imaging, radar, and video to audio—most of the data was collected using adult patients. In our research group, we focus on the development of a system that is especially suitable for children.

In a preclinical phase, we sought to develop a contactless system that can monitor respiratory movements. The core sensor technology of the system consists of a time-of-flight camera (ToF camera) and a 3D imaging module (i.e., hardware and software components for depth data acquisition and reconstruction) [13]. To process the raw depth data, software was implemented, and machine learning algorithms were trained on annotated datasets to reliably extract respiratory movement. These devices were trained to record thoracic and abdominal excursions to determine respiratory parameters such as breathing rate. An important aspect of the development process was the optimization of the AI-based region-of-interest (ROI) detection to ensure accurate measurement across different sleeping positions and conditions (e.g., body covered by a blanket). For further information on the technical solution, please refer to our previously published paper [13,14].

The next step was to evaluate the feasibility and clinical applicability of the prototype for contactless respiratory monitoring. Accordingly, the primary objective of this first validation study was to determine whether the system could accurately detect respiratory rate in pediatric patients. In addition, we explored the potential of the prototype to identify abnormal breathing patterns.

In the following, the terms “prototype” and “Quietam Nox” will be used interchangeably to refer to our novel system.

## 2. Material and Methods

### 2.1. Patients

The study involved 27 pediatric patients aged between 6 months and 12 years (Table 1). Patients were admitted to the pediatric sleep laboratory at the University Children’s Hospital, Regensburg (Bavaria). The recruitment strategy for the technical validation had two aims:

(1) To cover a broad range for the assessment of the clinical applicability of the prototype.
(2) Inclusion of respiratory pathologies, such as apneas, as well as patients without sleep-related disorders (i.e., normal examples). Therefore, we included children with central apnea and hypopnea, patients with other respiratory diagnoses, and children with no sleep-related disorders at all (Table 2). The patients’ characteristics are summarized in Table 1.

In all cases, the parents or caregivers had consented for their child to take part in the study. The study was approved by the Ethics Committee of the University of Regensburg, Germany, with the file number 20-1931-101.

### 2.2. Polysomnography (PSG)

All study participants underwent attended overnight polysomnography (Sleepware G3 Version 3.9.5, Philips Respironics, 1001 Murry Ridge Ln, Murrysville, PA, USA). Sleep airflow was measured using a standard nasal pressure cannula, and respiratory effort was recorded via a single channel (inductive thoracic effort belt; Philips Respironics). Sleep stages were assessed through continuous monitoring of the electroencephalogram, bilateral electrooculogram, and chin electromyogram. Pulse oximetry was recorded from a finger sensor (Masimo) with 1% saturation accuracy. Additional parameters included electrocardiogram, limb movements, and snoring sounds. An experienced sleep technician monitored participants remotely from a control room using signal displays, a microphone, and a closed-circuit television camera.

Sleep staging was performed according to the current American Academy of Sleep Medicine (AASM) pediatric scoring guidelines as outlined in the *AASM Scoring Manual, Version 3*, published on 15 February 2023.

### 2.3. Technical Test Setup

**Figure 1:**
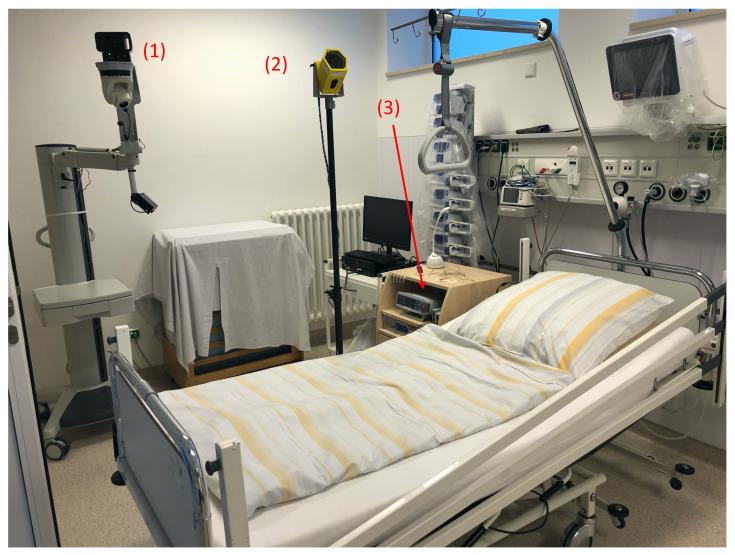
Test setup: (1) PSG camera; (2) time-of-flight camera; and (3) head box module.

The test setup was configured with a fixed setting (Figure 1). The camera’s angle was 40° relative to the horizontal plane. The camera was positioned at a height between 130 and 170 cm. The distance to the bed was selected to ensure that the patient was centered within the field of view of the camera. This resulted in a distance of 50–100 cm from the foot part of the bed.

To ensure diagnostic reliability in clinical settings, we tested the prototype under various conditions: Obstructed view: The prototype was assessed for accuracy when the child’s body was not covered, partially covered, or nearly completely covered by a blanket.

### 2.4. Data Acquisition, Analysis, and Algorithmic Processing with AI

To extract respiratory parameters from the raw depth data, a dedicated software pipeline was developed. The core of this pipeline consisted of an artificial-intelligence-based region-of-interest (ROI) detection algorithm that automatically localized the thoracic and abdominal areas within each frame. This ensured that only movement relevant to respiration was analyzed, thereby reducing the influence of unrelated body motion or environmental noise.

Following ROI detection, the temporal signal was processed using filtering and smoothing techniques to minimize motion artifacts. A moving-average filter was applied to the depth trajectories and respiratory cycles were subsequently identified by detecting periodic excursions within the ROI signal. For each 60 s segment, respiratory rate was computed by counting zero-crossings of the filtered curve. Respiratory rate was calculated as the integer number of respiratory cycles within fixed 60 s segments for both PSG and prototype signals. Consequently, differences between the two methods are inherently discrete and appear as integer values in the Bland–Altman analysis.

To generate data with minimal susceptibility to motion artifacts, analysis was restricted to recordings taken five minutes after entry into the deep sleep phase N3. After obtaining stable results in N3, the evaluation was extended to the REM sleep phase, which is likewise characterized by near motionlessness and therefore less prone to artifact contamination. Because of the large volume of data collected, only 60 s sections were used for the initial evaluation.

### 2.5. Statistical Analysis

Statistical comparison between the two monitoring methods was conducted using correlation analyses and Bland–Altman plots to assess agreement in respiratory rate measurements. The outcome was the concordance between the respiratory rates recorded by the prototype and PSG, respectively, with a predefined acceptance threshold of a 95% confidence interval for agreement.

## 3. Results

### 3.1. Qualitative Output

**Figure 2:**
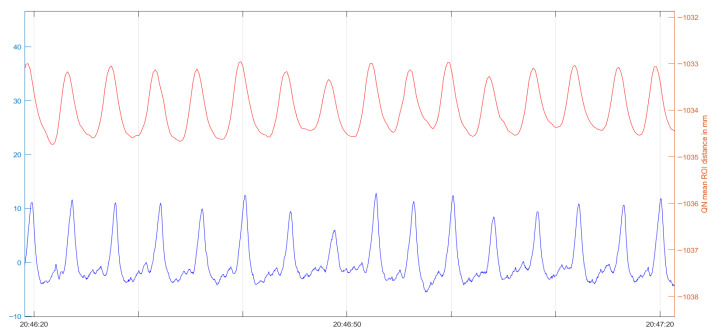
Graphical processing of the respiratory movement compared between Quietam Nox (orange) and conventional PSG (blue).

In total, 296 h of respiratory data were collected. For analysis, multiple 60 s segments were extracted. Respiratory rates were derived from thoracic and abdominal excursions. For improved comparability, the raw signals were subsequently smoothed using a moving-average filter. A representative curve of the raw data is presented in Figure 2 to illustrate the signal characteristics obtained from the prototype.

The thoracic and abdominal curves were superimposed, and the absolute number of zero-crossings within a 60 s interval was calculated. A meaningful signal is displayed, similar to that of conventional PSG belts.

### 3.2. Accuracy of Contactless Prototype for Respiratory Monitoring

**Figure 3:**
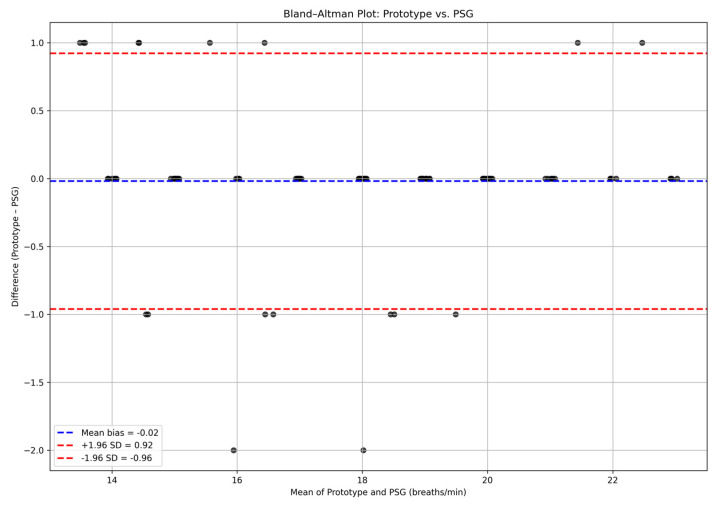
Bland–Altman Plot comparing Quietam Nox with conventional PSG. For visualization purposes, a small horizontal jitter was applied to overlapping data points; underlying values remain unchanged.

The Bland–Altman analysis showed minimal bias between the two methods, showing an accuracy of the prototype with a mean bias of −0.04 (Figure 3). Individual data points are displayed in the revised Bland–Altman plot, enabling visual assessment of agreement across subjects.

**Figure 4:**
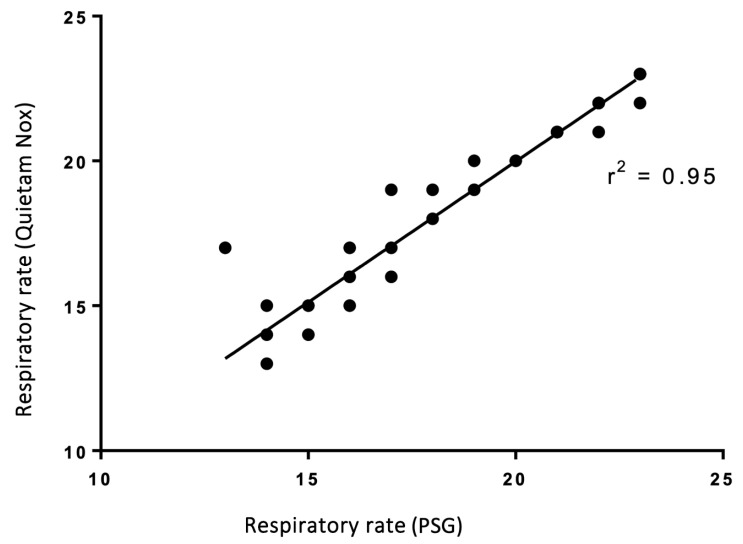
Coefficient of determination (R^2^) between the Quietam Nox prototype and conventional PSG. Fewer visible points appear due to overlapping values from identical respiratory rates. The correlation was statistically significant (*p* < 0.001).

The clinical evaluation of the contactless respiratory monitoring prototype demonstrated a high level of agreement in respiratory rate with the gold-standard PSG, as indicated by Bland–Altman analysis showing limits of agreement within −1 to +1 breaths per minute. Across 27 participants, the agreement between the prototype and PSG reached an R^2^ value of 0.95 (Figure 4). This includes data from across all age groups, from infants to older children. Although data from 27 patients were included, fewer visible points appear due to overlapping values from identical respiratory rates. The correlation was statistically significant (*p* < 0.001).

### 3.3. Performance in Different Sleep Positions and Conditions

**Figure 5:**
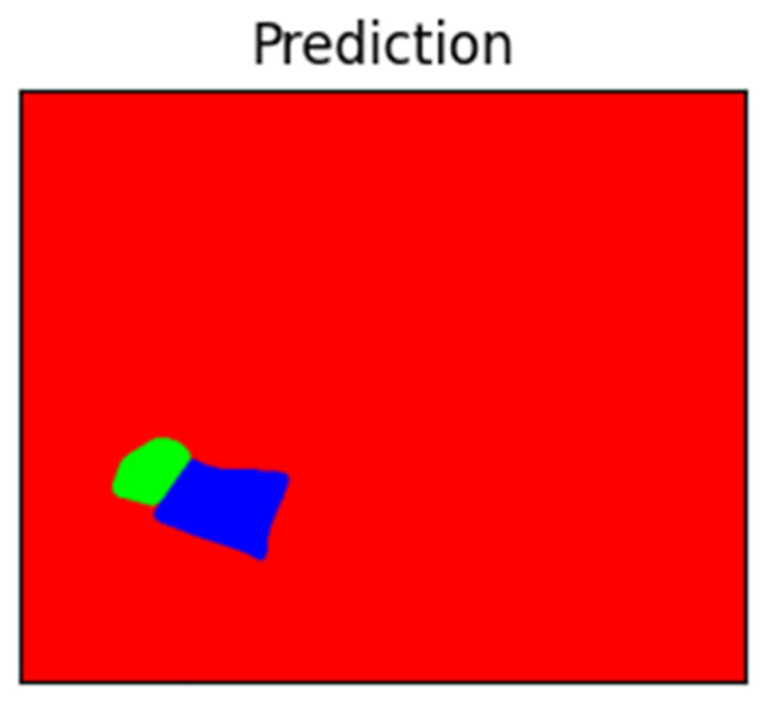
Four-dimensional visualization and prediction of the region of interest (ROI) with the patient’s head (green) and thoracoabdominal area (blue). Other areas were visualized as red.

The system’s ability to determine respiratory rate in different sleep scenarios was also evaluated. We found that the respiratory rate measured by our system showed a high correlation across different body positions, even when the child’s body was partially covered by a blanket. The prototype consistently delivered valid respiratory signals, with accurate respiratory rate detection even in scenarios where the body was partially covered by a sleeping bag, blanket, or romper (Figure 5).

### 3.4. Detection of Central Apneas

Segments containing apnea events were selected based on PSG findings for qualitative comparison. No systematic quantification of apnea frequency was performed. Some of the 27 children included in the study exhibited respiratory pathologies. The prototype detected deviations from normal respiratory patterns during the recordings. Among these events were episodes consistent with central apnea as well as periods of hypopnea.

**Figure 6:**
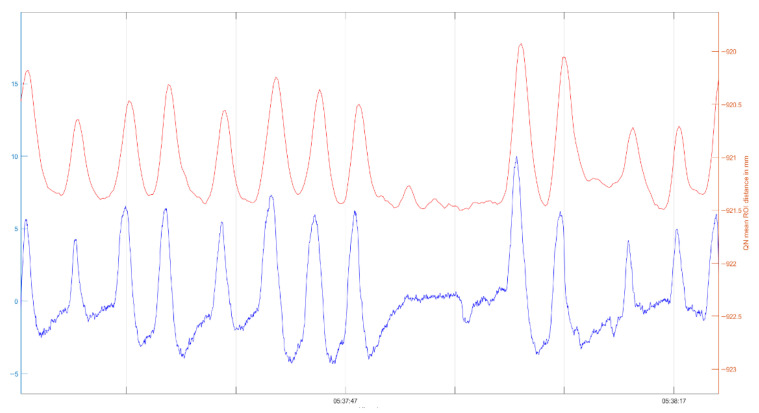
Graphical processing of the respiratory movement compared between Quietam Nox (orange) and conventional PSG (blue) showing central apnea.

The contactless system accurately captured the respiratory effort and frequency in children with respiratory disease (Figure 6; central apnea).

### 3.5. Technical Challenges and Solutions

During the early stages of clinical testing, technical challenges arose due to infrared interference between the 3D camera of the prototype and the infrared-based monitoring equipment used in the PSG. The time-of-flight camera’s signal exposure disturbed the PSG video. However, this issue was resolved with infrared filters and system reconfigurations, allowing for clear, uninterrupted data collections.

Another problem was that the curves of the time-of-flight camera appeared time-shifted and jagged. For graphical analysis, the data were manually synchronized using a frame-by-frame comparison, and a moving-average filter was applied for signal smoothing.

### 3.6. Usability and Feasibility

Feedback from the clinical staff and parents indicated that the system’s contactless nature was an improvement compared to the PSG effort belts, particularly in reducing patient discomfort and simplifying the monitoring process. The compact, contactless design was well-received, and its ease of use in a real-life clinical setting was confirmed during the study. However, in the present study, these aspects have not been evaluated systematically, e.g., using structured questionnaires for parents and health care staff.

## 4. Discussion

This study evaluated the clinical performance of a novel contactless respiratory monitoring prototype specifically designed for pediatric application. A key distinction of our system is its dedicated focus on children, in contrast to many previous approaches that primarily addressed adult populations. Earlier work using infrared thermography, radar-based systems, and camera-based approaches demonstrated feasibility primarily in adult populations, while pediatric data remain comparatively limited [1,3,4,5,15]. Our prototype was validated directly in a pediatric sleep laboratory, thereby targeting a patient group in which comfort and feasibility are of particular importance. The system reliably captured respiratory rates and patterns across various sleeping positions, even when thoracoabdominal movements were partially obscured by blankets or sleeping bags. This represents a frequent real-world scenario that other research groups have highlighted as a limitation of contactless monitoring. The integration of machine learning algorithms for automated ROI detection was an additional strength of our approach. This feature reduced irrelevant signal input and optimized the extraction of respiratory information, ultimately minimizing the data burden for analysis. Through in situ validation against PSG, the prototype demonstrated not only technical feasibility but also clinical applicability, thereby reinforcing its potential relevance for routine pediatric sleep diagnostics.

Beyond technical feasibility, the contactless design of the system may also carry broader clinical implications. By eliminating thoracic and abdominal belts, patient comfort will be enhanced and the potential disruption of natural sleep reduced. A further possible advantage, which requires systematic evaluation in future studies, is the mitigation of the “first night effect”. If sleep is less disturbed by monitoring devices, diagnostic accuracy could increase, while simultaneously improving efficiency in the sleep laboratory. In such a scenario, more patients might be accommodated within the same timeframe, thus optimizing the use of clinical resources.

It is important to emphasize that the prototype is not intended to replace full polysomnography. This perspective is consistent with current pediatric sleep medicine guidelines, which continue to regard PSG as the comprehensive reference standard for the evaluation of sleep-related breathing disorders in children [15]. Rather, it serves as a contactless alternative to conventional respiratory belts, while PSG remains essential for comprehensive sleep diagnostics.

The main strength of this work lies in the prospective validation of a contactless monitoring system in a real-world pediatric setting, directly compared with the current gold standard, PSG. The inclusion of patients across a broad pediatric age range, including infants, strengthens the generalizability of the findings. Furthermore, the prototype was evaluated under various realistic conditions, such as different sleeping positions and partial body coverage, which increases the clinical relevance of the results. Nevertheless, several limitations must be considered. The analysis was restricted to selected sleep stages (N3 and REM), which are characterized by limited movement. Full-night validation across all sleep stages, including N1 and N2, will be necessary to confirm robustness under more challenging conditions. Despite the integration of AI-based ROI detection, the large amount of raw data still required manual review and processing, limiting the immediate scalability of the system. Further development of automated analysis pipelines is therefore essential to reduce evaluation time and facilitate routine clinical use. Finally, the current prototype involves higher costs compared to conventional effort belts, mainly due to the time-of-flight technology. However, the rapid pace of technological development suggests that these costs are likely to decrease in the near future. Minor discrepancies between prototype- and PSG-derived respiratory rates primarily reflect methodological differences in breath counting within fixed 60 s windows rather than true measurement failures.

The prototype reliably detects respiratory motion and is therefore well suited for identifying central apnea events. However, differentiation of obstructive apnea and hypopnea requires airflow-based measurements, which are not included in the current system.

Taken together, this first clinical validation of our prototype provides evidence that contactless respiratory monitoring is feasible in pediatric patients and might yield reliable respiratory data under real-life clinical conditions. While additional technical improvements and larger-scale studies are required, particularly with full-night analyses, our findings highlight the promise of this approach as a less invasive and more child-friendly alternative to conventional PSG belts.

## Figures and Tables

**Table 1:** Patient characteristics (n = 27).

Characteristic	Value (n = 27)
Age, mean ± SD	3.15 ± 3.30 years
Age, range	0.21–16.13 years
Male sex, n (%)	18 (66.7%)
Female sex, n (%)	9 (33.3%)
Height, mean ± SD	89.7 ± 19.9 cm
Height, range	55–133 cm
Weight, mean ± SD	13.6 ± 5.9 kg
Weight, range	4.6–26.0 kg

**Table 2:** Respiratory events of all patients (n = 27).

Patient ID	RDI (Events/h)	Obstructive Apnea (Events/h)	Mixed Apnea (Events/h)	Central Apnea (Events/h)	Hypopnea (Events/h)
QN-1	4.10	1.00	1.00	18.00	13.00
QN-2	10.20	8.00	6.00	41.00	34.00
QN-3	9.15	1.50	2.00	29.00	21.50
QN-4	0.60	0.00	0.00	2.00	2.00
QN-5	0.80	0.00	0.00	7.00	1.00
QN-6	12.86	1.00	3.00	60.00	10.00
QN-7	2.90	0.00	0.00	5.00	23.00
QN-8	6.30	4.00	0.00	10.00	39.00
QN-9	13.30	0.00	5.00	69.00	23.00
QN-10	2.40	1.00	2.00	7.00	9.00
QN-11	1.60	0.00	0.00	4.00	9.00
QN-12	1.10	0.00	0.00	5.00	5.00
QN-13	10.70	2.00	4.00	69.00	7.00
QN-14	29.80	4.00	2.00	21.00	4.00
QN-15	2.00	1.00	0.00	4.00	9.00
QN-16	2.60	0.00	2.00	5.00	13.00
QN-17	9.60	0.00	2.00	13.00	63.00
QN-18	2.95	0.00	2.00	13.00	13.00
QN-19	10.00	3.00	3.00	13.00	70.00
QN-20	26.30	86.00	7.00	67.00	73.00
QN-21	30.20	23.00	2.00	197.00	22.00
QN-22	8.07	9.00	3.00	17.00	28.00
QN-23	10.30	0.00	0.00	43.00	3.00
QN-24	1.80	0.00	0.00	9.00	3.00
QN-25	4.10	0.00	0.00	12.00	21.00
QN-26	192.12	37.00	47.00	821.00	40.00
QN-27	19.00	0.00	0.00	3.00	79.00
Summary (median [range])	9.60 (0.60–192.12)	1.00 (0–86)	2.00 (0–47)	13.00 (2–821)	21.00 (1–79)

Values are presented as events per hour. Summary values are shown as median (range). RDI, respiratory disturbance index.

## Data Availability

The data presented in this study are not publicly available due to ethical and data protection restrictions related to pediatric patient data. Anonymized data may be made available from the corresponding author upon reasonable request.
